# Supplementary material for: Mnemonic construction and representation of temporal structure in the hippocampal formation
Source: Nat Commun. 2022 Jun 23;13:3395. doi: 10.1038/s41467-022-30984-3 (PMC9226117; doi:10.1038/s41467-022-30984-3)
Supplement: Supplementary file 1 — Supplementary Information [file 41467_2022_30984_MOESM1_ESM.pdf]

Supplemental Figures

Supplemental Figure 1

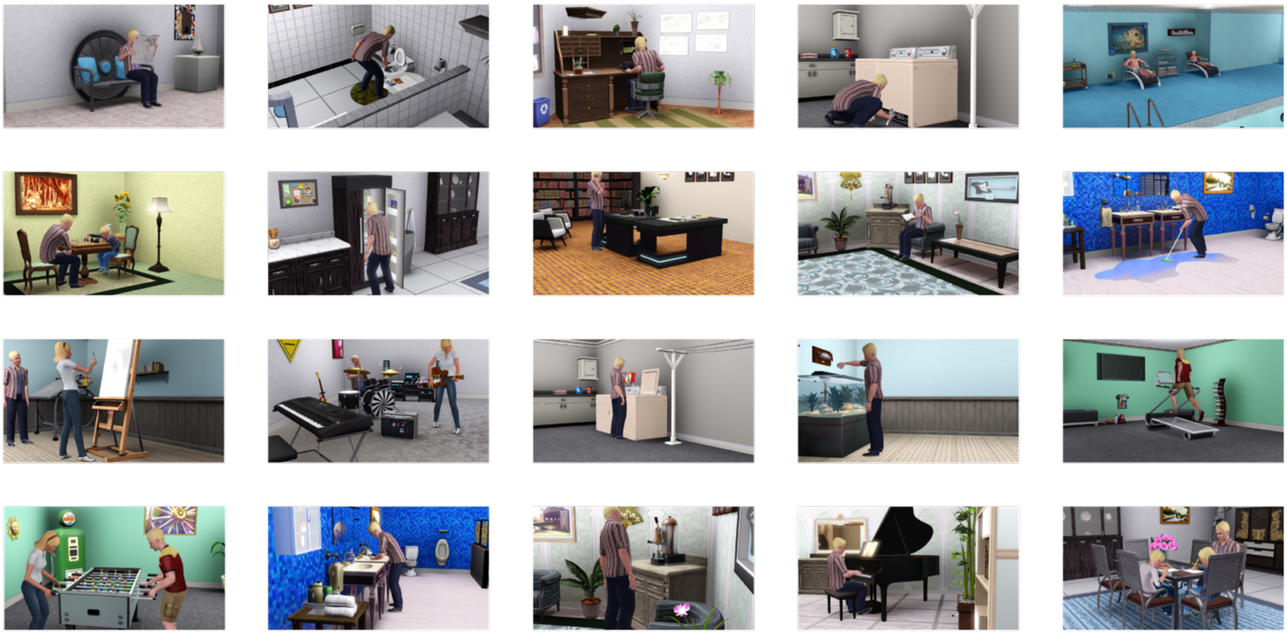

Supplemental Figure 1. Overview of the event images used as stimuli. All scenes were devoid of windows to exclude diurnal cues, such as shadows or light color, and were selected so they would be plausible at any time of day. For each participant, event images were randomly allocated to sequences and event times. Event images were created using the life-simulation computer game The Sims 3 (Electronic Arts). The Sims 3 and screenshots of it are licensed property of Electronic Arts, Inc. All rights reserved.

Supplemental Figure 2

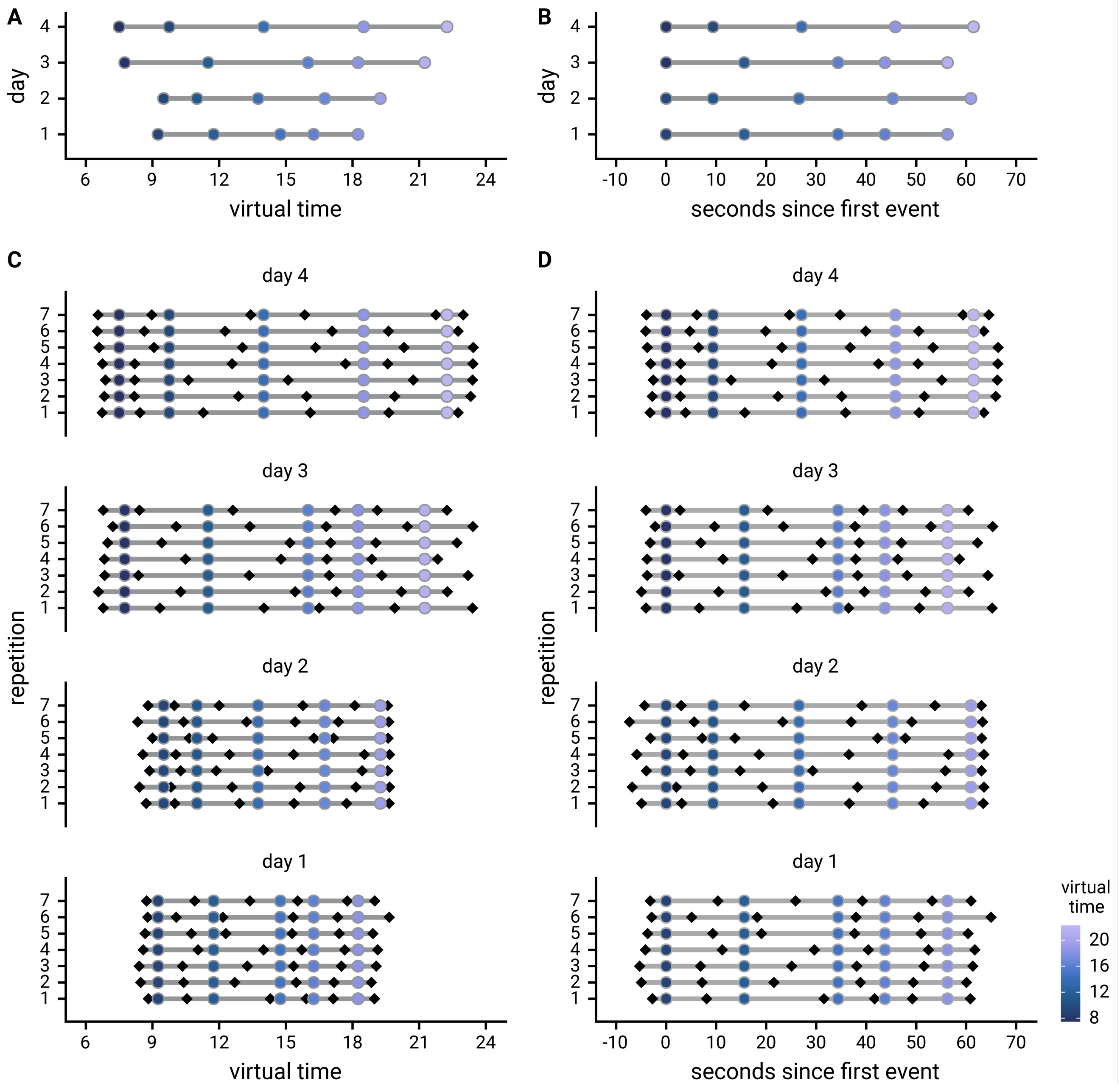

Supplemental Figure 2. Design of the day learning task. **A**. Each of the four virtual days consisted of a sequence of five events. Event sequences are shown in virtual time, i.e. relative to the hidden clock. Less virtual time passes within the bottom two sequences because clock speed was manipulated between sequences. **B**. Event sequences shown in real time relative to the first event. A comparable amount of real time (in seconds) elapses during each event sequence despite different amounts of virtual time passing. **C**, **D**. Sequences in virtual and real time as shown in (**A**) and (**B**), respectively, but separately for each of the seven repetitions of each sequence during the learning task. Black diamonds indicate the time cues shown to one randomly chosen example participant during the task. Time cues varied across repetitions and differed across participants.

Supplemental Figure 3

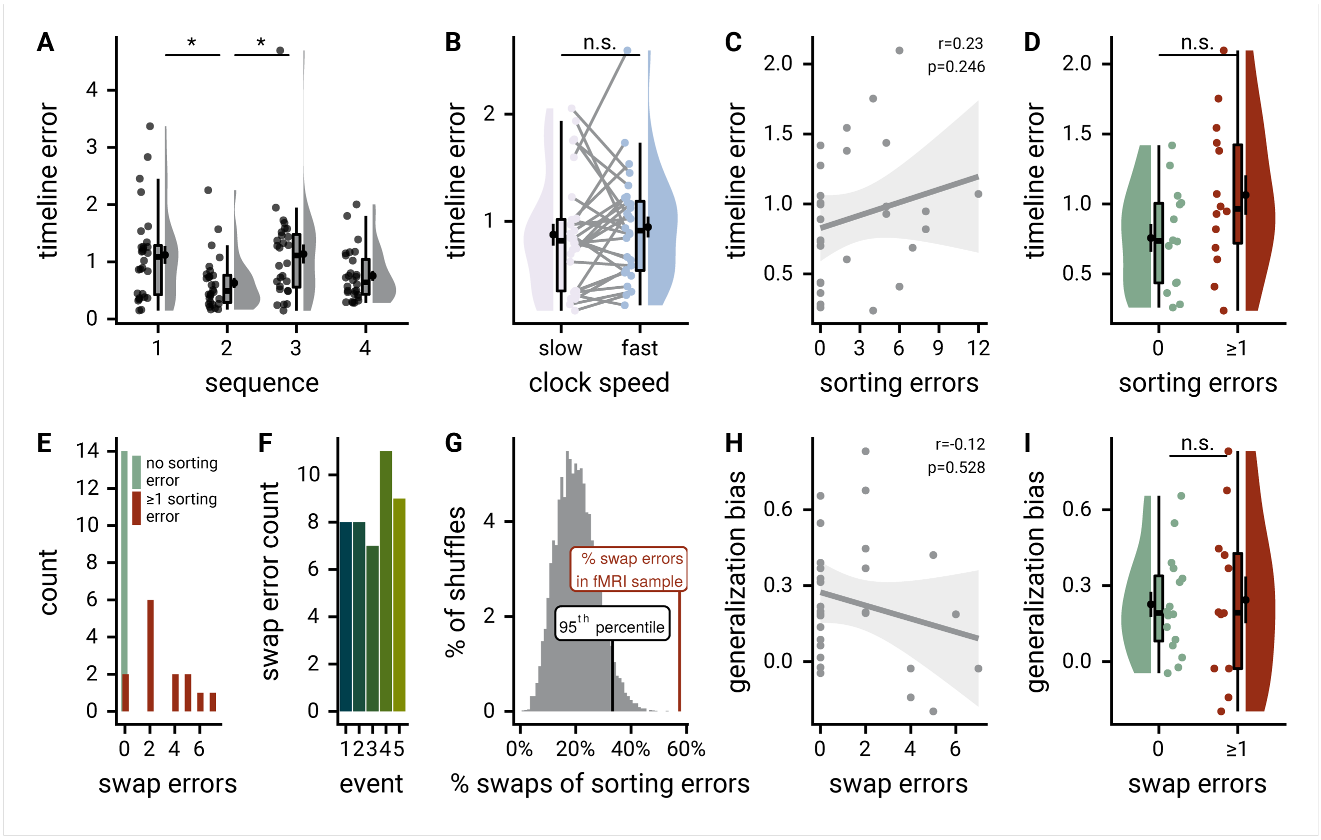

Supplemental Figure 3. Memory performance. A. A permutation-based repeated measures ANOVA revealed a significant effect of sequence on mean absolute errors in the timeline task ($F_{3,81}$=5.86, p<0.001, post hoc contrasts: sequence 1 vs. 2: t_{27}=3.38, p=0.001, sequence 1 vs. 3: t_{27}=-0.12, p=0.912, sequence 1 vs. 4: t_{27}=2.59, p=0.013, sequence 2 vs. 3: t_{27}=-2.92, p=0.001, sequence 2 vs. 4: t_{27}=-1.15, p=0.271, sequence 3 vs. 4: t_{27}=2.15, p=0.023). *p < Bonferroni-adjusted alpha-level of 0.008, corrected for 6 pairwise post hoc comparisons (two-tailed). **B**. Mean absolute timeline errors did not differ statistically between sequences with fast and slow clock speed (t_{27}=-0.82, p=0.423). **C**. The number of errors in the sorting task did not correlate with the mean absolute error in the timeline task across participants (r=0.23, p=0.246). **D**. Mean absolute errors in the timeline task were not statistically different between participants who made one or more errors (red) or no errors in the sorting task (green) in the sorting task (two-tailed t-test for independent samples, t_{26}=-1.79, p=0.085). **E**. Histogram shows the number of swap errors for participants with (red) and without (green) errors in the sorting task. **F**. The distribution of swap errors over sequence positions did not deviate statistically from uniformity ($\chi^2(1)$=1.07, p=0.899). **G**. Histogram shows the null distribution of the proportion of swap errors expected under random sorting errors. The proportion of swap errors observed in our sample (red line) exceeded the 95th percentile of the null distribution (black line). **H**. The number of swap errors was not significantly correlated with the generalization bias (Spearman r=0.12, p=0.528). **I**. The generalization bias in the timeline task was not significantly different between participants who made one or more swap errors (red) or no swap errors (green) in the sorting task (t_{26}=0.18, p=0.861). **A**, **B**, **D**, **I**. Circles show data from n=28 participants; boxplots show median and upper/lower quartile along with whiskers extending to most extreme data point within 1.5 interquartile ranges above/below the upper/lower quartile; black circles with error bars correspond to mean±S.E.M.; distributions show probability density function of data points. **C**, **H**. Each circle shows data from one participant, grey line and shaded region indicate least squares line and confidence interval.

Supplemental Figure 4

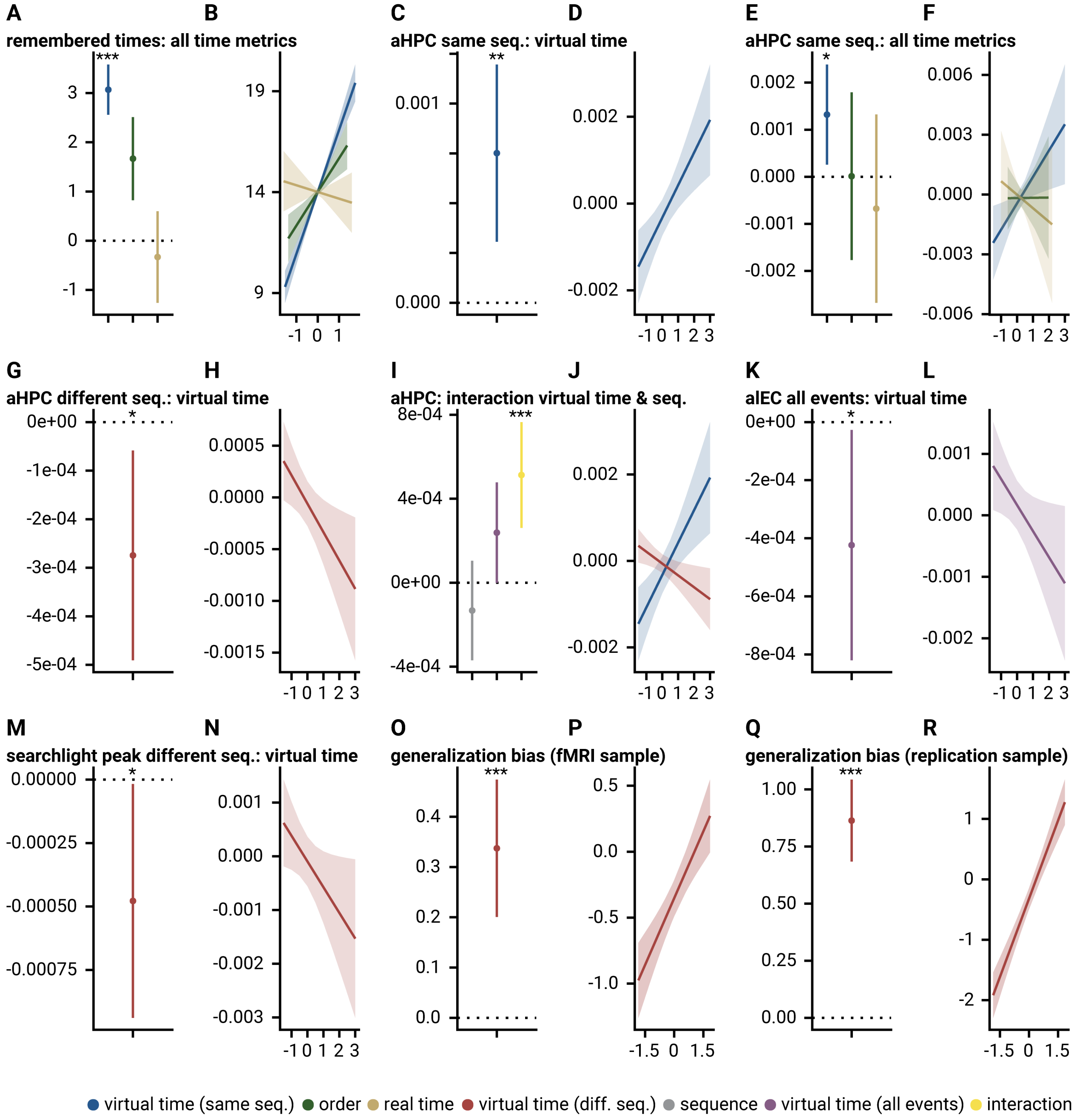

Supplemental Figure 4. Mixed model results. A, C, E, G, I, K, M, O, Q. Dot plots show parameter estimates and 95% confidence intervals for fixed effects of mixed model analyses. **B, D, F, H, J, L, N, P, R.** Line plots show estimated marginal means with 95% confidence intervals. **A**, **B**. Remembered times in the time line task are predicted by virtual event times with order and real time in the model (c.f**. Figure 2B**). **C**, **D**. Temporal distances in virtual time explain representational change in the anterior hippocampus (aHPC) for same-sequence events (c.f. **Figure 4B**). **E**, **F**. Temporal distances in virtual time explain representational change in the aHPC for same-sequence events when competing for variance with temporal distances based on order and real time (c.f. **Figure 4D**). **G**, **H**. Temporal distances in virtual time explain representational change in the aHPC for different-sequence events (c.f. **Figure 5A**). **I**, **J**. There was a significant interaction of virtual temporal distances and sequence membership characterized by a differential relationship between temporal distances and aHPC representational change for event pairs from the same sequence or from different sequences (c.f. **Figure 5A**). **K**, **L**. Virtual temporal distances explain representational change in the anterior-lateral entorhinal cortex (alEC) when collapsing across all event pairs (c.f. **Figure 6B**). **M, N**. In the aHPC peak cluster of the same-sequence searchlight analysis, virtual temporal distances were siginificantly related to representational change for events from different sequences (c.f. **Figure 7B**). **O-R**. The relative time of events from other sequences predicted signed event time construction errors as measured in the timeline task (c.f. **Figure 8CD**) in the main fMRI sample (**O**, **P**) and in the independent replication sample (**Q**, **R**).

Supplemental Figure 5

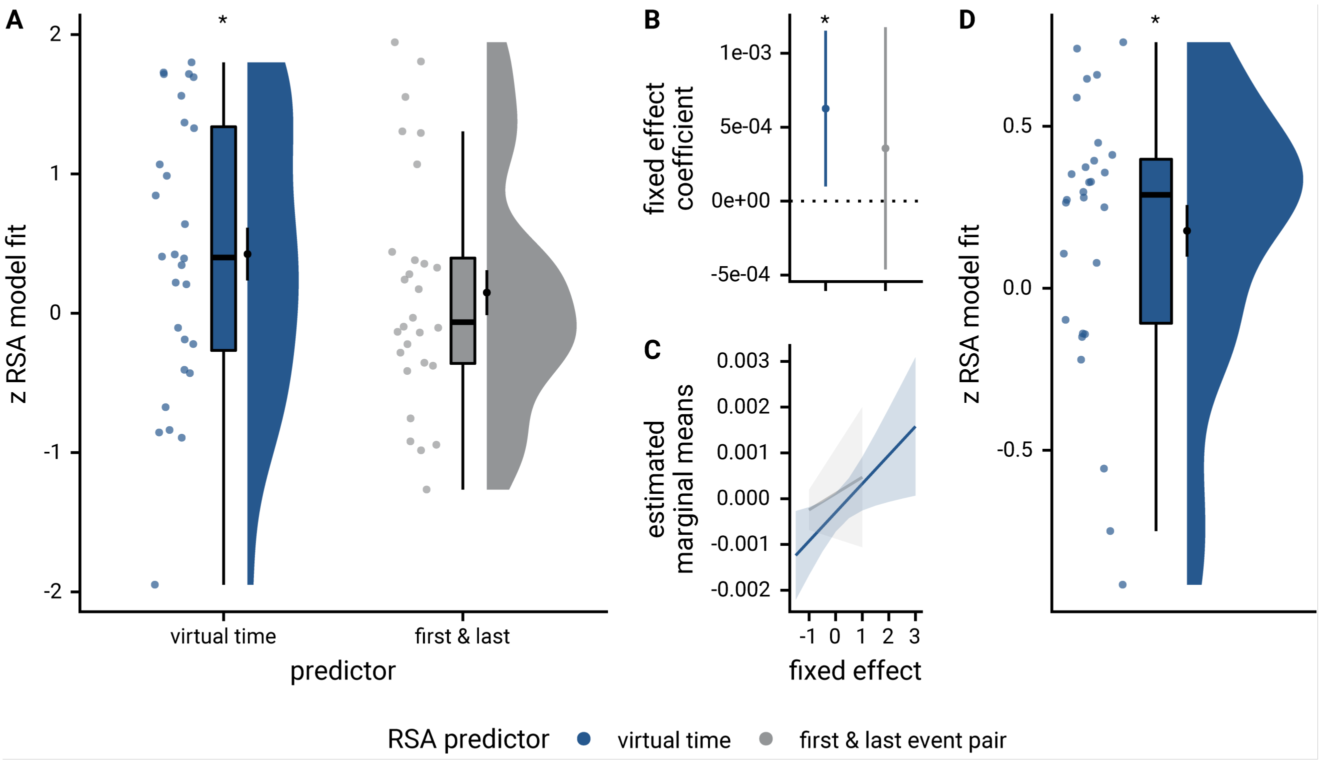

Supplemental Figure 5. The relationship of virtual time and hippocampal pattern similarity change is not driven by the first and last event of a sequence. A. Z-values from the summary statistics approach show a significant positive effect of virtual time on pattern similarity change in the anterior hippcampus when competing for variance with a control predictor of no interest accounting for variance explained by whether pairs of events were made up from the first and last event of a sequence or not. **B, C.** Fixed effect estimate with 95% confidence intervals (**B**) and estimated marginal means (**C**) visualize the results of the corresponding mixed model. **D**. We implemented participant-specific regression analyses with order and real time distances as predictors of hippocampal pattern similarity change. The plot shows a significant effect of virtual temporal distances when tested on the residuals of these linear models. Thus, variance that cannot be explained by the other time metrics can be accounted for by virtual temporal distances. This analysis was conducted only using the summary statistics approach because the residuals of a mixed model are more difficult to interpret than those of participant-specific regression analyses using ordinary least squares. **A, D.** Circles show Z-values from the summary statistics approach for n=28 participants; boxplot shows median and upper/lower quartile along with whiskers extending to most extreme data point within 1.5 interquartile ranges above/below the upper/lower quartile; black circle with error bars corresponds to mean±S.E.M.; distribution shows probability density function of data points. * $p<0.05$

Supplemental Figure 6

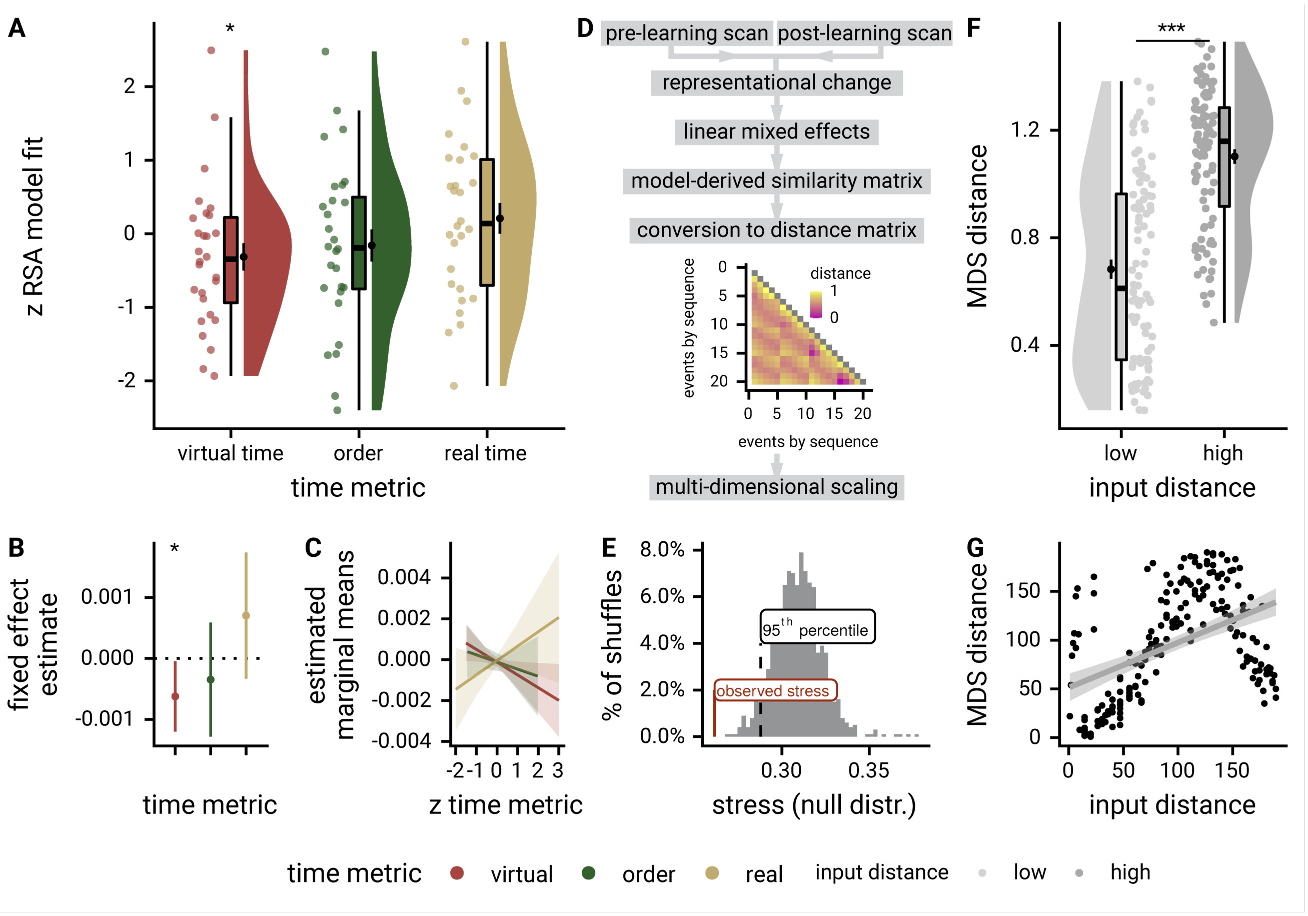

Supplemental Figure 6. Virtual time predicts hippocampal pattern similarity change for events from different sequences. A. Z-values show the relationship of the different time metrics to representational change in the anterior hippocampus based on participant-specific multiple regression analyses for pairs of events from different sequences. Circles show participant-specific Z-values from summary statistics approach for n=28 participants; boxplot shows median and upper/lower quartile along with whiskers extending to most extreme data point within 1.5 interquartile ranges above/below the upper/lower quartile; black circle with error bars corresponds to mean±S.E.M.; distribution shows probability density function of data points. **B**, **C**. Parameter estimates with 95% confidence intervals (**B**) and estimated marginal means (**C**) show the fixed effects of the three time metrics from the corresponding mixed model. * p<0.05 after exclusion of one outlier excluded based on the boxplot criterion. **D.** A linear mixed model capturing the interaction effect of virtual temporal distances and sequence membership (**Figure 5, Supplemental Figure 4IJ**) was fitted to hippocampal representational change. An event-by-event similarity matrix was derived from the fixed effects of this model. Similarities were converted distances and then used as input for multidimensional scaling (see Methods). **E**. The stress value observed in the MDS analysis (red line) was significantly smaller than the 5th percentile (black dashed line) of a surrogate distribution of stress values obtained from shuffling the dissimilarities before running MDS in each of 1000 iterations. **F**. Pairs of events separated by a large distance in the input distance matrix were separated by a larger Euclidean distance in the resulting MDS configuration (t_{188}=9.35, p<0.001, d=1.35, 95% CI [1.03, 1.67]). *** p <0.001. Circles show data from n=190 pairwise temporal distances between events; boxplot shows median and upper/lower quartile along with whiskers extending to most extreme data point within 1.5 interquartile ranges above/below the upper/lower quartile; black circle with error bars corresponds to mean±S.E.M.; distribution shows probability density function of data points. **G**. There was a significant Spearman correlation of input distances and MDS configuration distances (r=0.46, p<0.001), but visual inspection reveals a non-linear relationship where very high distances are systematically underestimated in the MDS configuration. This is likely because the data were projected onto only two dimensions for visualization. More dimensions would be needed to improve the fit of the MDS configuration and the input distance matrix. Distances are shown as ranks because non-metric MDS was used (high ranks for high distances). Line shows least squares line, shaded region corresponds to 95% confidence interval.

Supplemental Figure 7

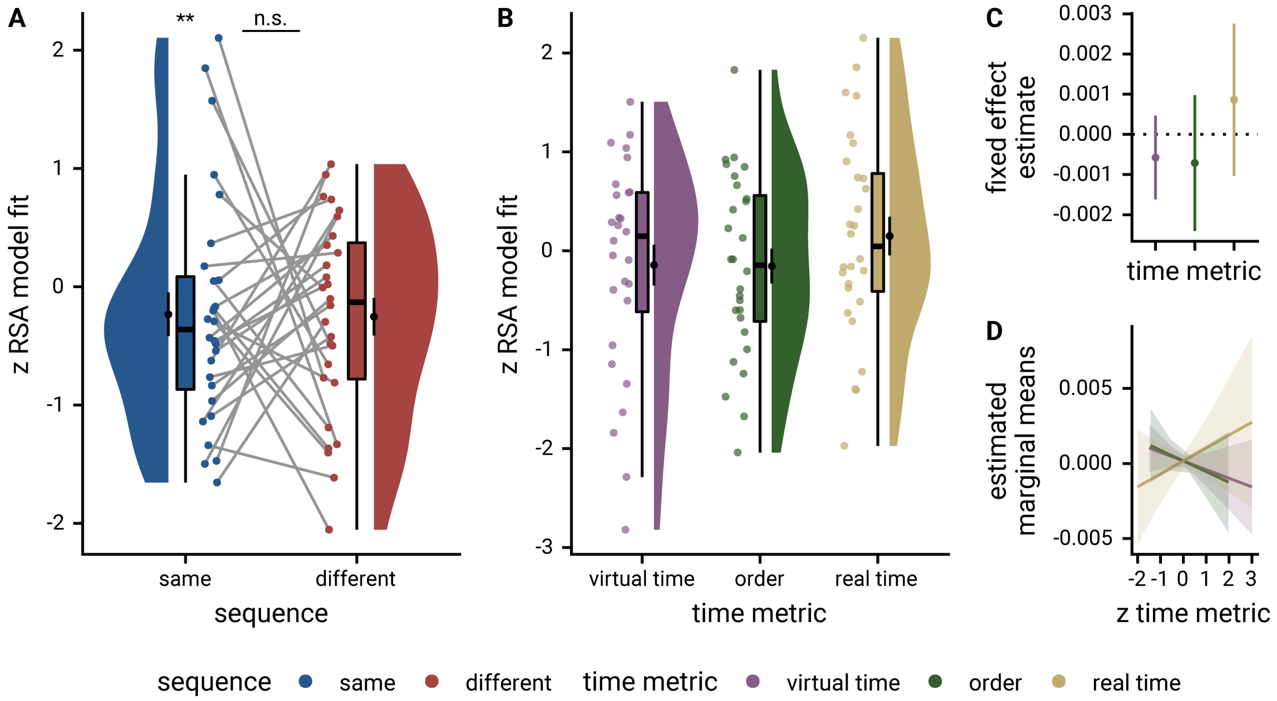

Supplemental Figure 7. Pattern similarity change in the anterior-lateral entorhinal cortex. A. Relationship of pattern similarity change and temporal distances between events from the same and different sequences in the anterior-lateral entorhinal cortex. There was no statistically significant difference between correlations of virtual temporal distances and representational change in the anterior-lateral entorhinal cortex depending on whether event pairs were from the same or different sequences. Entorhinal representational change was negatively related to temporal distances between events from the same sequence (summary statistics: t_{24}=-3.54, p=0.002, d=-0.69, 95% CI [-1.17, -0.27]; α=0.025, corrected for separate tests of events of the same and different sequences; three outliers excluded based on the boxplot criterion). The relationship between entorhinal pattern similarity change for events from different sequences was not statistically different from zero (summary statistics: t_{27}=-1.60, p=0.122, d=-0.29, 95% CI [-0.69, 0.08]; α=0.025, corrected for separate tests of events of the same and different sequences). ** p<0.01 after outlier exclusion. **B**. Z-values show the relationship of the different time metrics to representational change in the anterior-lateral entorhinal cortex based on participant-specific multiple regression analyses. Analysis includes all pairs of events. **C**, **D**. Parameter estimates with 95% confidence intervals (**C**) and estimated marginal means (**D**) show the fixed effects of the three time metrics from the corresponding mixed model. **A, B**. Circles show Z-values from summary statistics approach for n=28 participants; boxplot shows median and upper/lower quartile along with whiskers extending to most extreme data point within 1.5 interquartile ranges above/below the upper/lower quartile; black circle with error bars corresponds to mean±S.E.M.; distribution shows probability density function of data points.

Supplemental Figure 8

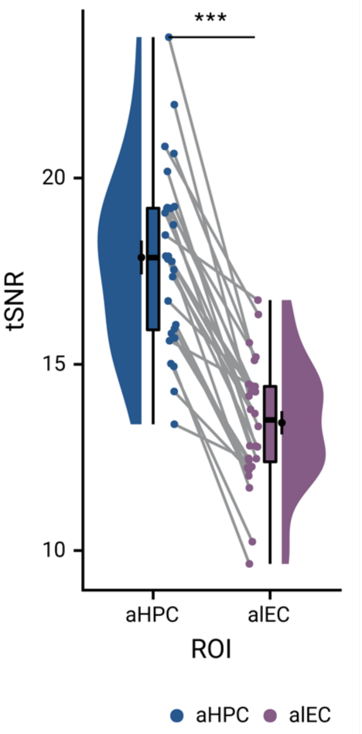

Supplemental Figure 8. Temporal signal-to-noise ratio in the anterior hippocampus and the anterior-lateral entorhinal cortex. A. The temporal signal-to-noise ratio was quantified as the mean unsmoothed signal over time divided by its standard deviation. It was calculated for each voxel and then averaged across voxels in a region of interest. The temporal signal-to-noise ratio was higher in the anterior hippocampus (aHPC) than in the anterior-lateral entorhinal cortex (alEC, summary statistics: $t_{27}=12.43$, $p<0.001$, $d=1.99$, 95% CI [1.65, 3.13]). Circles show data from n=28 participants; boxplot shows median and upper/lower quartile along with whiskers extending to most extreme data point within 1.5 interquartile ranges above/below the upper/lower quartile; black circle with error bars corresponds to mean±S.E.M.; distribution shows probability density function of data points. *** p<0.001

Supplemental Figure 9

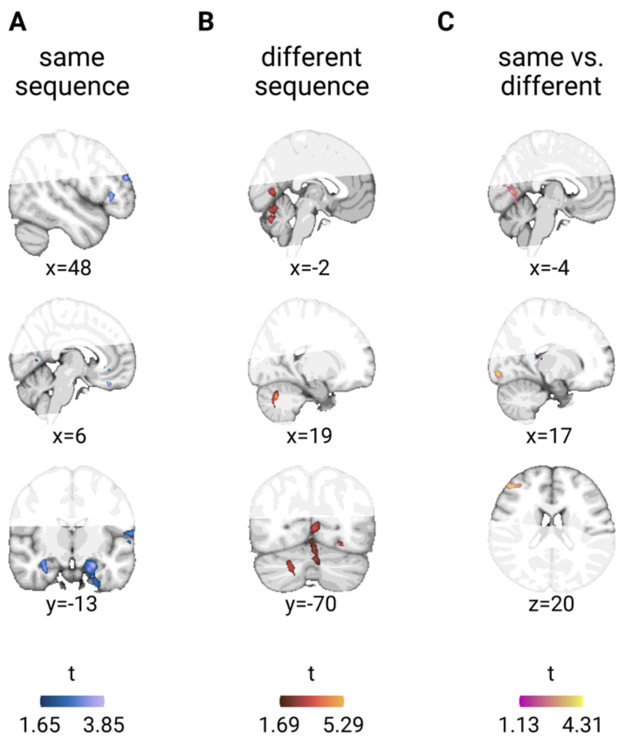

Supplemental Figure 9. Exploratory searchlight results. A. For same-sequence event pairs, clusters of voxels in which pattern similarity change correlated positively with temporal distances were detected in the frontal pole, frontal medial cortex and left entorhinal cortex (see Supplemental Table 12). **B.** Pattern similarity change correlated negatively with temporal distances between events from different sequences in the cerebellum and lingual gyrus (see Supplemental Table 14). **C.** The interaction effect, defined as correlations of temporal distances and pattern similarity change depending on whether pairs of events belonged to the same sequence or not, was observed in the occipital pole, lingual gyrus, frontal pole, temporal fusiform cortex and the intracalcerine sulcus (see Supplemental Table 15). **A-C.** Statistical images are thresholded at p<0.01 uncorrected for display purposes. No clusters outside the hippocampal-entorhinal region survived corrections for multiple comparisons.

Supplemental Figure 10

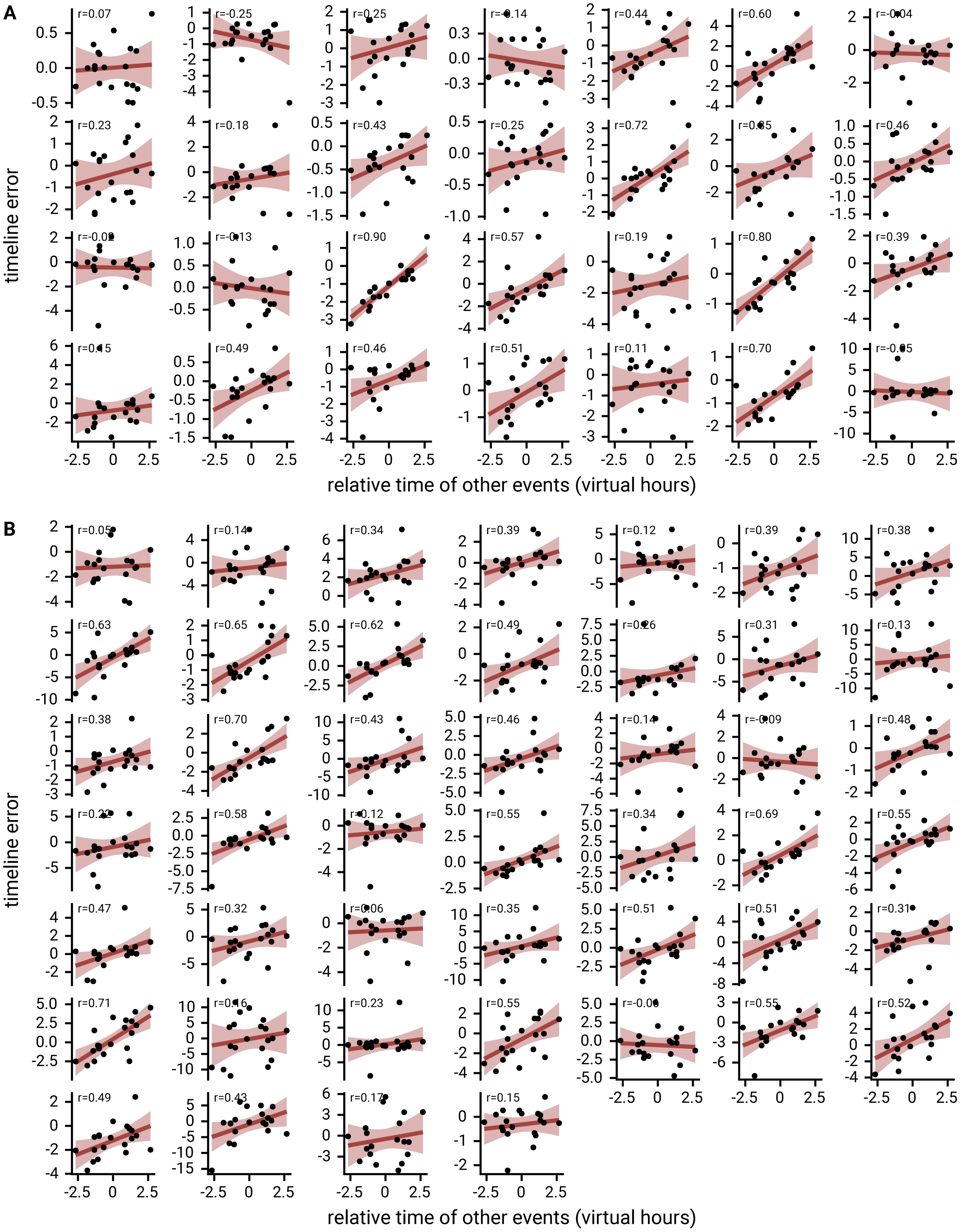

Supplemental Figure 10. Generalization bias in individual participants. A, B. Each panel shows the data from one participant. Each circle corresponds to one event. The x-axis indicates the average relative time of the events occupying the same sequence position in other sequences. The y-axis shows the signed error of constructed event times as measured in the timeline task. The regression line and its confidence interval are overlaid in red. Positive slopes of the regression line indicate that constructed event times are biased by the average time of events in the other sequences. Correlation coefficients are based on Pearson correlation. **A** shows data from the main sample; **B** from the replication sample.

Supplemental Tables

Supplemental Table 1

Mixed Model: Virtual time explains constructed times with order and real time in the model

fixed effects						
**term**		**estimate**	**SE**	**t-value**	**95% CI**	
intercept		14.010019	0.069962	200.25	13.868056	14.151981
virtual time		3.069324	0.259967	11.81	2.558874	3.579774
order		1.667630	0.430230	3.88	0.822785	2.512476
real time		-0.332261	0.473306	-0.70	-1.261696	0.597173
**random effects**						
**group**	**term**					**estimate**
participant	intercept					0.221991
participant	virtual time (SD)					0.232089
participant	correlation random intercepts and random slopes					0.165592
residual	SD					1.324919
**model comparison**						
**model**	**npar**	**AIC**	**LL**	**χ^2**	**df**	**p**
reduced model	7	2053.90	-1019.95			
full model	8	1939.95	-961.98	115.95	1	4.88e-27

model: memory_time~virtual_time_z+order_z+real_time_z+(1+virtual_time_z|sub_id);
SE: standard error, CI: confidence interval, SD: standard deviation, npar: number of parameters, LL: log likelihood, df: degrees of freedom, corr.: correlation

Supplemental Table 2

Mixed Model: Virtual time explains representational change for same-sequence events in the anterior hippocampus

**fixed effects**						
**term**		**estimate**	**SE**	**t-value**	**95% CI**	
intercept		-0.000326	0.000211	-1.54	-0.000740	0.000088
virtual time		0.000751	0.000220	3.42	0.000307	0.001196
**random effects**						
**group**	**term**					**estimate**
participant	intercept (SD)					0.000001
participant	virtual time					0.000257
residual	SD					0.006917
**model comparison**						
**model**	**npar**	**AIC**	**LL**	**χ^2**	**df**	**p**
reduced model	4	-7943.56	3975.78			
full model	5	-7951.43	3980.72	9.87	1	0.002

model: ps_change~vir_time_diff+((1|sub_id)+(0+vir_time_diff|sub_id));
SE: standard error, CI: confidence interval, SD: standard deviation, npar: number of parameters, LL: log likelihood, df: degrees of freedom, corr.: correlation

Supplemental Table 3

Mixed Model: Virtual time explains representational change for same-sequence events in the anterior hippocampus when controlling for the effect of first-last event pairs

**fixed effects**						
**term**		**estimate**	**SE**	**t-value**	**95% CI**	
intercept		-0.000015	0.000421	-0.04	-0.000841	0.000810
virtual time		0.000626	0.000264	2.37	0.000099	0.001152
first-last pair		0.000357	0.000418	0.85	-0.000462	0.001176
**random effects**						
**group**	**term**					**estimate**
participant	intercept (SD)					0.000001
participant	virtual time (SD)					0.000258
residual	SD					0.006914
**model comparison**						
**model**	**npar**	**AIC**	**LL**	**χ^2**	**df**	**p**
reduced model	5	-7946.81	3978.40			
full model	6	-7950.16	3981.08	5.36	1	0.021

model: ps_change~vir_time_diff+first_last+((1|sub_id)+(0+vir_time_diff|sub_id));
SE: standard error, CI: confidence interval, SD: standard deviation, npar: number of parameters, LL: log likelihood, df: degrees of freedom, corr.: correlation

Supplemental Table 4

Mixed Model: Virtual time explains representational change for same-sequence events in the anterior hippocampus when including order and real time in the model

**fixed effects**						
**term**		**estimate**	**SE**	**t-value**	**95% CI**	
intercept		-0.000281	0.000219	-1.28	-0.000711	0.000149
virtual time		0.001321	0.000541	2.44	0.000258	0.002383
order		0.000012	0.000908	0.01	-0.001768	0.001793
real time		-0.000676	0.001019	-0.66	-0.002675	0.001323
**random effects**						
**group**	**term**					**estimate**
participant	virtual time (SD)					0.000260
residual	SD					0.006913
**model comparison**						
**model**	**npar**	**AIC**	**LL**	**χ^2**	**df**	**p**
reduced model	5	-7946.84	3978.42			
full model	6	-7950.76	3981.38	5.92	1	0.015

model: ps_change~vir_time_diff+order_diff+real_time_diff+(0+vir_time_diff|sub_id);
SE: standard error, CI: confidence interval, SD: standard deviation, npar: number of parameters, LL: log likelihood, df: degrees of freedom, corr.: correlation

Supplemental Table 5

Mixed Model: Virtual time explains representational change for different-sequence events in the anterior hippocampus

**fixed effects**						
**term**		**estimate**	**SE**	**t-value**	**95% CI**	
intercept		-0.000061	0.000110	-0.55	-0.000276	0.000155
virtual time		-0.000275	0.000110	-2.51	-0.000491	-0.000058
**random effects**						
**group**	**term**					**estimate**
participant	virtual time (SD)					0.000000
residual	SD					0.007107
**model comparison**						
**model**	**npar**	**AIC**	**LL**	**χ^2**	**df**	**p**
reduced model	3	-29621.39	14813.69			
full model	4	-29625.40	14816.70	6.01	1	0.014

model: ps_change~vir_time_diff+(0+vir_time_diff|sub_id);
SE: standard error, CI: confidence interval, SD: standard deviation, npar: number of parameters, LL: log likelihood, df: degrees of freedom, corr.: correlation

Supplemental Table 6

Mixed Model: Virtual time explains representational change for different-sequence events in the anterior hippocampus when including order and real time in the model

**fixed effects**						
**term**		**estimate**	**SE**	**t-value**	**95% CI**	
intercept		-0.000101	0.000112	-0.90	-0.000319	0.000118
virtual time		-0.000623	0.000294	-2.12	-0.001201	-0.000046
order		-0.000348	0.000478	-0.73	-0.001284	0.000589
real time		0.000702	0.000529	1.33	-0.000334	0.001739
**random effects**						
**group**	**term**					**estimate**
participant	virtual time (SD)					0.000000
residual	SD					0.007077
**model comparison**						
**model**	**npar**	**AIC**	**LL**	**χ^2**	**df**	**p**
reduced model	5	-28594.64	14302.32			
full model	6	-28597.12	14304.56	4.48	1	0.034

model: ps_change~vir_time_diff+order_diff+real_time_diff+(0+vir_time_diff|sub_id);
SE: standard error, CI: confidence interval, SD: standard deviation, npar: number of parameters, LL: log likelihood, df: degrees of freedom, corr.: correlation

Supplemental Table 7

Mixed Model: The effect of virtual time differs between same-sequence and different-sequence events in the anterior hippocampus

**fixed effects**						
**term**		**estimate**	**SE**	**t-value**	**95% CI**	
intercept		-0.000193	0.000121	-1.60	-0.000430	0.000044
virtual time		0.000238	0.000122	1.95	-0.000001	0.000478
day		-0.000133	0.000121	-1.10	-0.000370	0.000104
interaction virtual time and day		0.000513	0.000127	4.05	0.000261	0.000765
**random effects**						
**group**	**term**					**estimate**
participant	interaction virtual time and day (SD)					0.000176
residual	SD					0.007066
**model comparison**						
**model**	**npar**	**AIC**	**LL**	**χ^2**	**df**	**p**
reduced model	5	-37569.38	18789.69			
full model	6	-37581.75	18796.87	14.37	1	1.50e-04

model: ps_change~vir_time_diff*same_day_dv+(0+vir_time_diff:same_day_dv|sub_id);
SE: standard error, CI: confidence interval, SD: standard deviation, npar: number of parameters, LL: log likelihood, df: degrees of freedom, corr.: correlation

Supplemental Table 8

Mixed Model: The effect of virtual time differs between same-sequence and different-sequence events in the anterior hippocampus when including interactions with other time metrics

**fixed effects**					
**term**	**estimate**	**SE**	**t-value**	**95% CI**	
intercept	-0.000190	0.000122	-1.55	-0.000428	0.000049
virtual time	0.000237	0.000124	1.92	-0.000005	0.000480
day	-0.000130	0.000121	-1.07	-0.000368	0.000108
interaction virtual time and day	0.000769	0.000262	2.93	0.000255	0.001283
interaction order and day	0.000287	0.000418	0.69	-0.000533	0.001106
interaction real time and day	-0.000558	0.000464	-1.20	-0.001468	0.000351

**random effects**		
**group**	**term**	**estimate**
participant	interaction virtual time and day (SD)	0.000176
residual	SD	0.007065

**model comparison**						
**model**	**npar**	**AIC**	**LL**	**χ^2**	**df**	**p**
reduced model	7	-37572.96	18793.48			
full model	8	-37579.53	18797.77	8.57	1	0.003

model: ps_change~vir_time_diff*same_day_dv+order_diff:same_day_dv+real_time_diff:same_day_dv+(0+vir_time_diff:same_day_dv|sub_id);
SE: standard error, CI: confidence interval, SD: standard deviation, npar: number of parameters, LL: log likelihood, df: degrees of freedom, corr.: correlation

Supplemental Table 9

Mixed Model: Virtual time explains representational change in the anterior-lateral entorhinal cortex (all events)

fixed effects						
**term**		**estimate**	**SE**	**t-value**	**95% CI**	
intercept		0.000167	0.000202	0.83	-0.000229	0.000563
virtual time		-0.000424	0.000202	-2.09	-0.000820	-0.000027
**random effects**						
**group**	**term**					**estimate**
participant	virtual time (SD)					0.000000
residual	SD					0.014734
**model comparison**						
**model**	**npar**	**AIC**	**LL**	χ^2	**df**	**p**
reduced model	3	-29767.39	14886.69			
full model	4	-29769.77	14888.89	4.39	1	0.036

model: ps_change~vir_time_diff+(0+vir_time_diff|sub_id);
SE: standard error, CI: confidence interval, SD: standard deviation, npar: number of parameters, LL: log likelihood, df: degrees of freedom, corr.: correlation

Supplemental Table 10

Mixed Model: Virtual time does not explain representational change for different-sequence events in the anterior-lateral entorhinal cortex when including order and real time in the model

**fixed effects**						
**term**		**estimate**	**SE**	**t-value**	**95% CI**	
intercept		0.000167	0.000202	0.83	-0.000229	0.000563
virtual time		-0.000576	0.000531	-1.09	-0.001617	0.000464
order		-0.000712	0.000862	-0.83	-0.002402	0.000978
real time		0.000862	0.000966	0.89	-0.001031	0.002754
**random effects**						
**group**	**term**					**estimate**
participant	virtual time (SD)					0.000000
residual	SD					0.014733
**model comparison**						
**model**	**npar**	**AIC**	**LL**	**χ^2**	**df**	**p**
reduced model	5	-29767.41	14888.71			
full model	6	-29766.59	14889.30	1.18	1	0.278

model: ps_change~vir_time_diff+order_diff+real_time_diff+(0+vir_time_diff|sub_id);
SE: standard error, CI: confidence interval, SD: standard deviation, npar: number of parameters, LL: log likelihood, df: degrees of freedom, corr.: correlation

Supplemental Table 11

Mixed Model: The effect of virtual time differentially depends on sequence membership in the anterior hippocampus and the anterior-lateral entorhinal cortex

**fixed effects**					
**term**	**estimate**	**SE**	**t-value**	**95% CI**	
intercept	-0.000193	0.000219	-0.89	-0.000622	0.000235
virtual time	0.000238	0.000200	1.19	-0.000153	0.000630
day	-0.000133	0.000197	-0.67	-0.000520	0.000254
ROI	0.000455	0.000279	1.63	-0.000093	0.001002
virtual time * day	0.000513	0.000202	2.54	0.000117	0.000909
virtual time * ROI	-0.000810	0.000282	-2.87	-0.001363	-0.000257
day * ROI	0.000261	0.000279	0.94	-0.000286	0.000808
virtual time * day * ROI	-0.000745	0.000294	-2.54	-0.001321	-0.000169

**random effects**		
**group**	**term**	**estimate**
participant	intercept (SD)	0.000496
participant	corr. intercept, virtual time:day:ROI1	-1.000000
participant	corr. intercept, virtual time:day:ROI-1	-0.151340
participant	virtual time:day:ROI1 (SD)	0.000170
participant	corr. virtual time:day:ROI1, virtual time:day:ROI-1	0.151340
participant	virtual time:day:ROI-1 (SD)	0.000421
residual	SD	0.011540

**model comparison**						
**model**	**npar**	**AIC**	**LL**	χ^2	**df**	**p**
reduced model	14	-64699.87	32363.94			
full model	15	-64704.19	32367.09	6.31	1	0.012

model: ps_change~vir_time_diff*same_day_dv*roi_dv+(1+vir_time_diff:same_day_dv:roi_dv|sub_id);
SE: standard error, CI: confidence interval, SD: standard deviation, npar: number of parameters, LL: log likelihood, df: degrees of freedom, corr.: correlation

Supplemental Table 12

Searchlight Analysis: Virtual time explains representational change for same-sequence events

Atlas Label	Voxel Extent	x	y	z	COG x	COG y	COG z	t	p
**Searchlight results in a priori regions of interest, p-values corrected using small volume correction**									
left hippocampus	193	-24	-13	-20	-23.3	-13.1	-19.8	4.53	0.006
right hippocampus	96	31	-16	-20	30.1	-16.7	-19.8	3.56	0.035
left hippocampus	76	-27	-20	-15	-27.9	-19.5	-16.6	3.47	0.029
**Exploratory searchlight results, p-values uncorrected**									
**Atlas Label**	**Voxel Extent**	**x**	**y**	**z**	**COG x**	**COG y**	**COG z**	**t**	**p**
frontal pole	399	50	44	16	48.3	41.6	19.2	3.96	0.0002
frontal pole	173	53	41	-7	51.1	42.9	-4.45	4.56	0.0002
left entorhinal cortex	119	-18	-16	-32	-21.2	-14.6	-31.2	3.45	0.0004
inferior frontal gyrus	91	40	27	2	44.2	28	3.59	4.29	0.0002
lingual gyrus	86	-17	-58	-15	-15.7	-56.9	-9.64	3.82	0.0002
frontal medial cortex	49	7	35	-23	6.29	36.7	-24.1	4.28	0.0004

x, y, z refer to MNI coordinates of minimum p-value in cluster, t denotes the most extreme t-value, COG: center of gravity

Supplemental Table 13

Mixed Model: Virtual time explains representational change for different-sequence events in the peak cluster of the same-sequence searchlight analysis

**fixed effects**						
**term**		**estimate**	**SE**	**t-value**	**95% CI**	
intercept		-0.000097	0.000234	-0.41	-0.000557	0.000362
virtual time		-0.000478	0.000234	-2.04	-0.000939	-0.000018
**random effects**						
**group**	**term**					**estimate**
participant	virtual time (SD)					0.000000
residual	SD					0.015162
**model comparison**						
**model**	**npar**	**AIC**	**LL**	**χ^2**	**df**	**p**
reduced model	3	-23257.87	11631.93			
full model	4	-23260.00	11634.00	4.13	1	0.042

model: ps_change~vir_time_diff+(0+vir_time_diff|sub_id);
SE: standard error, CI: confidence interval, SD: standard deviation, npar: number of parameters, LL: log likelihood, df: degrees of freedom, corr.: correlation

Supplemental Table 14

Searchlight Analysis: Virtual time explains representational change for different-sequence events

Exploratory searchlight results, p-values uncorrected									
**Atlas Label**	**Voxel Extent**	**x**	**y**	**z**	**COG x**	**COG y**	**COG z**	**t**	**p**
cerebellum	314	19	-68	-34	19.1	-66.3	-29.6	-5.37	0.0002
cerebellum	104	-1	-68	-14	-1.86	-69.1	-14.3	-3.44	0.0002
lingual gyrus	100	-1	-70	4	-2.68	-70.5	4.56	-3.73	0.0002

x, y, z refer to MNI coordinates of minimum p-value in cluster, t denotes the most extreme t-value, COG: center of gravity

Supplemental Table 15

Searchlight Analysis: Interaction of virtual time and sequence membership

Atlas Label	Voxel Extent	x	y	z	COG x	COG y	COG z	t	p
**Searchlight results in a priori regions of interest, p-values corrected using small volume correction**									
left hippocampus	359	-26	-20	-15	-23.4	-15.5	-18.6	4.15	0.014
right hippocampus	335	31	-16	-21	30.7	-15.1	-20.1	4.25	0.007
**Exploratory searchlight results, p-values uncorrected**									
**Atlas Label**	**Voxel Extent**	**x**	**y**	**z**	**COG x**	**COG y**	**COG z**	**t**	**p**
occipital pole	103	17	-91	-8	17.7	-90.6	-6.62	4.08	0.0002
lingual gyrus	102	-5	-73	5	-3.59	-70.4	5.01	3.72	0.0002
frontal pole	96	43	43	18	45.4	43.4	19.7	4.31	0.0006
frontal pole	45	35	43	17	37	43.2	18.5	3.81	0.0006
temporal fusiform cortex	40	-25	-10	-45	-25.3	-10.3	-42.9	3.14	0.0004
intracalcarine sulcus	33	-4	-77	11	-2.85	-75.8	11.5	3.56	0.0002

x, y, z refer to MNI coordinates of minimum p-value in cluster, t denotes the most extreme t-value, COG: center of gravity

Supplemental Table 16

Mixed Model: Behavioral generalization bias

fixed effects					
**term**	**estimate**	**SE**	**t-value**	**95% CI**	
intercept	-0.352481	0.069962	-5.04	-0.494444	-0.210518
relative time other events	0.337262	0.067360	5.01	0.200579	0.473945

random effects		
**group**	**term**	**estimate**
participant	intercept	0.220016
participant	relative time other events (SD)	-0.114173
participant	correlation random intercepts and random slopes	0.183681
residual	SD	1.331485

model comparison						
**model**	**npar**	**AIC**	**LL**	**χ^2**	**df**	**p**
reduced model	5	1958.57	-974.29			
full model	6	1942.67	-965.34	17.90	1	2.32e-05

model: timeline_error~rel_time_other_events_z+(1+rel_time_other_events_z|sub_id);
SE: standard error, CI: confidence interval, SD: standard deviation, npar: number of parameters, LL: log likelihood, df: degrees of freedom, corr.: correlation

Supplemental Table 17

Mixed Model: Behavioral generalization bias (replication)

**fixed effects**						
**term**		**estimate**	**SE**	**t-value**	**95% CI**	
intercept		-0.320564	0.089155	-3.60	-0.495488	-0.145640
relative time other events		0.863631	0.091472	9.44	0.684152	1.043110
**random effects**						
**group**	**term**					**estimate**
participant	relative time other events (SD)					0.000000
residual	SD					2.704218
**model comparison**						
**model**	**npar**	**AIC**	**LL**	**χ^2**	**df**	**p**
reduced model	3	4501.04	-2247.52			
full model	4	4449.30	-2220.65	53.74	1	2.29e-13

model: timeline_error~rel_time_other_events_z+(0+rel_time_other_events_z|sub_id);
SE: standard error, CI: confidence interval, SD: standard deviation, npar: number of parameters, LL: log likelihood, df: degrees of freedom, corr.: correlation
