# Supplementary material for: Mnemonic construction and representation of temporal structure in the hippocampal formation
Source: Nat Commun. 2022 Jun 23;13:3395. doi: 10.1038/s41467-022-30984-3 (PMC9226117; doi:10.1038/s41467-022-30984-3)
Supplement: Supplementary file 3 — Reporting Summary [file 41467_2022_30984_MOESM3_ESM.pdf]

nature portfolio

Corresponding author(s): Jacob L. S. Bellmund

Last updated by author(s): Apr 7, 2022

Reporting Summary

Nature Portfolio wishes to improve the reproducibility of the work that we publish. This form provides structure for consistency and transparency in reporting. For further information on Nature Portfolio policies, see our Editorial Policies and the Editorial Policy Checklist.

Statistics

For all statistical analyses, confirm that the following items are present in the figure legend, table legend, main text, or Methods section.

n/a	Confirmed	
☐	☒	The exact sample size (*n*) for each experimental group/condition, given as a discrete number and unit of measurement
☐	☒	A statement on whether measurements were taken from distinct samples or whether the same sample was measured repeatedly
☐	☒	The statistical test(s) used AND whether they are one- or two-sided<br>*Only common tests should be described solely by name; describe more complex techniques in the Methods section.*
☐	☒	A description of all covariates tested
☐	☒	A description of any assumptions or corrections, such as tests of normality and adjustment for multiple comparisons
☐	☒	A full description of the statistical parameters including central tendency (e.g. means) or other basic estimates (e.g. regression coefficient) AND variation (e.g. standard deviation) or associated estimates of uncertainty (e.g. confidence intervals)
☐	☒	For null hypothesis testing, the test statistic (e.g. *F*, *t*, *r*) with confidence intervals, effect sizes, degrees of freedom and *P* value noted<br>*Give P values as exact values whenever suitable.*
☒	☐	For Bayesian analysis, information on the choice of priors and Markov chain Monte Carlo settings
☐	☒	For hierarchical and complex designs, identification of the appropriate level for tests and full reporting of outcomes
☐	☒	Estimates of effect sizes (e.g. Cohen's *d*, Pearson's *r*), indicating how they were calculated

Our web collection on statistics for biologists contains articles on many of the points above.

Software and code

Policy information about availability of computer code

Data collection	The tasks inside the MR scanner were presented on a rear-projection screen with a resolution of 800x600 pixels and implemented using Presentation (version 16.2, Neurobehavioral Systems). Subsequently, outside of the scanner, participants performed two short memory tasks in front of a computer screen, implemented with custom Matlab code.
Data analysis	Data analysis was carried out using FSL (version 5.0.4) and R (version 3.6.1). Region of interest (ROI) masks were based on participant-specific FreeSurfer segmentations (version 6.0.0-2) and the Harvard-Oxford atlas distributed with FSL (version 5.04.). Linear mixed models were implemented using lme4 (version 1.1-23). Analysis code and documentation are available on GitHub (https://jacbel.github.io/virtem_code/).

For manuscripts utilizing custom algorithms or software that are central to the research but not yet described in published literature, software must be made available to editors and reviewers. We strongly encourage code deposition in a community repository (e.g. GitHub). See the Nature Portfolio guidelines for submitting code & software for further information.

Data

Policy information about availability of data

All manuscripts must include a data availability statement. This statement should provide the following information, where applicable:

- Accession codes, unique identifiers, or web links for publicly available datasets
- A description of any restrictions on data availability
- For clinical datasets or third party data, please ensure that the statement adheres to our policy

Data to reproduce the statistical analyses reported in this paper are available on the Open Science Framework (https://osf.io/zxnc8/). Source data are provided with this paper. Analysis code and documentation are available on GitHub (https://jacbel.github.io/virtem_code/).

March 2021

Field-specific reporting

Please select the one below that is the best fit for your research. If you are not sure, read the appropriate sections before making your selection.

- [x] Life sciences
- [ ] Behavioural & social sciences
- [ ] Ecological, evolutionary & environmental sciences

For a reference copy of the document with all sections, see nature.com/documents/nr-reporting-summary-flat.pdf

Life sciences study design

All studies must disclose on these points even when the disclosure is negative.

Sample size	31 participants were recruited for this experiment. No power calculation was performed a priori. The sample size followed the institute's common practices at the time of data acquisition (c.f. Milivojevic et al., Current Biology, 2015; Deuker et al., eLife, 2016).
Data exclusions	One participant aborted the experiment due to feeling claustrophobic when entering the MR scanner. Two participants were excluded from further analysis due to bad memory performance and technical difficulties during data acquisition. Thus, the final sample consisted of 28 participants.
Replication	To replicate the behavioral generalization bias, we conducted the same analysis in an independent group of participants. These participants (n=46) constituted the control groups of a behavioral experiment testing the effect of stress induction on temporal memory (Montijn et al, bioRxiv, 2021). They underwent the same learning task as described above with the only difference being the duration of this learning phase (4 rather than 7 mini-blocks of training). The timeline task was administered on the day after learning. The procedures are described in detail in Montijn et al. (bioRxiv, 2021). The data from this independent sample are shown in Figure 8D and Supplemental Figures 4QR and 10B.
Randomization	One group of participants was tested in the fMRI experiment and thus participants were not assigned to experimental groups.
Blinding	Participants were not assigned to experimental groups.

Reporting for specific materials, systems and methods

We require information from authors about some types of materials, experimental systems and methods used in many studies. Here, indicate whether each material, system or method listed is relevant to your study. If you are not sure if a list item applies to your research, read the appropriate section before selecting a response.

Materials & experimental systems

n/a	Involved in the study	
x		Antibodies
x		Eukaryotic cell lines
x		Palaeontology and archaeology
x		Animals and other organisms
	x	Human research participants
x		Clinical data
x		Dual use research of concern

Methods

n/a	Involved in the study	
x		ChIP-seq
x		Flow cytometry
	x	MRI-based neuroimaging

Human research participants

Policy information about studies involving human research participants

Population characteristics	The final sample of the fMRI experiment consisted of 28 participants (21 female, age: mean±standard deviation 23.04±3.21 years, range 18-31 years).
Recruitment	Participants were recruited via the online study recruitment tool of the Donders Institute for Brain Cognition & Behavior (The Netherlands). We are not aware of any selection biases (self or others) that could have impacted the results.
Ethics oversight	All proceedings were approved by the local ethics committee (CMO Regio Arnhem-Nijmegen).

Note that full information on the approval of the study protocol must also be provided in the manuscript.

Magnetic resonance imaging

Experimental design

Design type	event-related task-based design

Design specifications

In the picture viewing tasks (Figure 1B), participants viewed a stream of the event images. The task consisted of 10 mini-blocks. In each mini-block, the target image and the 20 images, which would later make up the virtual days (see Day Learning Task), were shown in random order. Mini-blocks were separated by breaks of 15 s. Stimulus presentations lasted 2.5 s and were time-locked to fMRI volume acquisition onsets. Scene stimuli within a mini-block were separated by 2 or 3 repetition times (TR), randomly assigned so that both stimulus onset asynchronies occurred equally often.

Behavioral performance measures

In the picture viewing task, participants' task was to look at the images attentively and to respond via button press whenever a target picture, which showed the father feeding the family's dog, was presented (pre-learning: 95.71% ±7.90% mean±standard deviation of percentage of hits; 881.34ms±131.43ms mean±standard deviation of average reaction times; post-learning: 95.71%±6.90% mean±standard deviation of percentage of hits; 841.40ms±162.16ms mean±standard deviation of average reaction times).

Acquisition

Imaging type(s)

Functional and structural MRI

Field strength

3T

Sequence & imaging parameters

MRI data were recorded with a 3T Siemens Skyra scanner (Siemens, Erlangen, Germany). A high-resolution 2D EPI sequence was used for functional scanning (TR=2270 ms, TE=24 ms, 40 slices, distance factor 13%, flip angle 85°, field of view (FOV) 210x210x68 mm, voxel size 1.5 mm isotropic). The field of view (FOV) was aligned to fully cover the medial temporal lobe, parts of ventral frontal cortex and (if possible) calcarine sulcus. Functional images for the two picture viewing tasks and the learning task were acquired in three runs. In addition to these partial-volume acquisitions, 10 scans of a functional whole-brain sequence were also acquired to improve registration during preprocessing. The sequence settings were identical to the functional sequence above, but instead of 40 slices, 120 slices were acquired, leading to a longer TR (6804.1ms). A structural scan was acquired for each participant (TR = 2300 ms; TE = 315 ms; flip angle = 8°; in-plane resolution = 256x256 mm; number of slices = 224, voxel resolution = 0.8x0.8x0.8 mm). Lastly, a gradient field map was acquired (for n = 21 participants only due to time constraints), with a gradient echo sequence (TR = 1020 ms; TE1 = 10 ms; TE2 = 12.46 ms; flip angle = 90°; volume resolution = 3.5x3.5x2 mm; FOV = 224x224 mm).

Area of acquisition

Structural MRI: wholebrain
Functional MRI: The field of view (FOV) was aligned to fully cover the medial temporal lobe, parts of ventral frontal cortex and (if possible) calcarine sulcus.

Diffusion MRI ☐ Used ☒ Not used

Preprocessing

Preprocessing software

Preprocessing was performed using FSL FEAT (version 6.00).

Normalization

Functional images from the two picture viewing tasks were then registered to the preprocessed mean image of the whole-brain functional scan. The whole-brain functional images were registered to the individual structural scans. The structural scans were in turn normalized to the MNI template (1-mm resolution). Gray matter segmentation was done on the structural images, and the results were mapped back to the space of the whole-brain functional scan for later use in the analysis.

Normalization template

MNI 1mm as distributed with FSL

Noise and artifact removal

Functional scans from the picture viewing tasks and the whole-brain functional scan were submitted to motion correction and high-pass filtering using FSL FEAT.

Volume censoring

Out of brain voxels were excluded.

Statistical modeling & inference

Model type and settings

Representational similarity analysis (RSA)109 was first implemented separately for the pre- and post-learning picture viewing task. It was carried out in ROIs co-registered to the whole-brain functional image and in searchlight analyses (see below). For the ROI analyses, preprocessed data were intersected with the participant-specific anterior hippocampus and anterolateral entorhinal cortex ROI masks as well as a brain mask obtained during preprocessing (only voxels within the brain mask in all mini-blocks were analyzed) and the gray matter mask. For each voxel within the ROI mask, motion parameters from FSL MCFLIRT were used as predictors in a general linear model (GLM) with the voxel time series as the dependent variable. The residuals of this GLM (i.e. data that could not be explained by motion) were taken to the next analysis step. As the presentation of images in the picture viewing tasks was locked to the onset of a new volume (see above), the second volume after image onset was selected for every trial, effectively covering the time between 2270 and 4540 ms after stimulus onset. Only data for the 20 event images that were shown in the learning task were analyzed; data for the target stimulus were discarded. The similarity between the multi-voxel activity pattern for every event image in every mini-block with the pattern of every other event in every other mini-block was quantified using Pearson correlation coefficients. Thus, comparisons of scenes from the same mini-block were excluded. Next, we calculated mean, Fisher z-transformed correlation coefficients for every pair of events, yielding separate matrices of pattern similarity estimates for the pre- and the post-learning picture viewing tasks (Figure 3).

Effect(s) tested

Summary Statistics Approach
In the summary statistics approach, we used the different time metrics as predictors for pattern similarity change. We set up a GLM with the given variable from the day learning task as a predictor and the pairwise representational change values as

the criterion for every participant. The t-values of the resulting model coefficients were then compared to a null distribution obtained from shuffling the dependent variable of the linear model (i.e. pattern similarity change) 10,000 times. This approach to permutation-testing of regression coefficients controls Type I errors even under situations of collinear regressors106. Resulting p-values for each coefficient were transformed to a Z-score. The Z-scores were then used for group-level inferential statistics.

Group-level statistics were carried out using permutation-based procedures. For t-tests, we compared the observed t-values against a surrogate distribution obtained from 10,000 random sign-flips to non-parametrically test against 0 or to assess within-participant differences between conditions (two-sided tests; α=0.05 unless stated otherwise). We report Cohen's d with Hedges' correction and its 95% confidence interval as computed using the effsize-package for R. For paired tests, Cohen's d was calculated using pooled standard deviations and confidence intervals are based on the non-central t-distribution. Permutation-based repeated measures ANOVAs were carried out using the permuco-package107 and we report generalized η2 as effect sizes computed using the afex-package108.

Linear Mixed Effects

Second, we employed linear mixed models to assess how learned sequence relationships were reflected in pattern similarity change using the lme4 package109. Mixed models have the advantage of estimating fixed effects and their interactions using all data, rather than performing inferential statistics on just one value per participant. We used the different time metrics as the fixed effects of interest. Factorial predictors (region of interest: anterior hippocampus and anterior-lateral entorhinal cortex; sequence: same vs. different) were deviation-coded. Within-subject dependencies were captured using random effects. Following the recommendation by Barr et al.102, we always first attempted to fit a model with a maximal random effects structure including random intercepts and random slopes for participants. If these models did not converge or resulted in singular fits, we reduced the random effects structure. We always kept random slopes for the fixed effect of interest in the model to avoid anti-conservativity when testing fixed effects or their interactions102,110. The mixed effects models were fitted using maximum likelihood estimation.

Specify type of analysis: ☐ Whole brain ☐ ROI-based ☒ Both

Anatomical location(s): Our previous work demonstrates representations reflecting the temporal relations of events from one sequence in the anterior hippocampus21 and the anterior-lateral entorhinal cortex27. More generally, these regions have been implicated in temporal coding and memory (for review, see10). Further, the hippocampus has been linked to inferential reasoning and generalization46,48,49,51,53. We thus focused our analyses on these regions. Region of interest (ROI) masks were based on participant-specific FreeSurfer segmentations (version 6.0.0-2), which yielded masks for the entire hippocampus and entorhinal cortex. These were co-registered to participants' functional space. We defined anterior hippocampus using the Harvard-Oxford atlas mask (thresholded at 50% probability), selecting all voxels anterior to MNI y=-21 based on Poppenk et al.98. The resulting anterior hippocampus mask was also co-registered to participants' functional space and intersected with the participant-specific hippocampal mask from FreeSurfer. The mask for the anterior-lateral entorhinal cortex was based on Navarro Schröder et al.99. It was co-registered to participants' functional space and intersected with the entorhinal cortex mask from FreeSurfer.

Statistic type for inference (See Eklund et al. 2016): ROI-based RSA: permutation-based significance tests and mixed models
RSA searchlights: threshold-free cluster enhancement (TFCE)

Correction: Bonferroni (ROI analyses), Searchlight: FWE based on FSL Randomise (small-volume corrected for voxels in a priori regions of interest)

Models & analysis

n/a	Involved in the study
☒	☐ Functional and/or effective connectivity
☒	☐ Graph analysis
☒	☐ Multivariate modeling or predictive analysis
